# High physical activity in persons with psoriatic arthritis is associated with reduced visceral fat mass and percentage body fat: the Trøndelag Health study

**DOI:** 10.1007/s00296-023-05348-9

**Published:** 2023-06-05

**Authors:** Abdirizak Ali Osman, Mari Hoff, Vibeke Videm

**Affiliations:** 1 Department of Clinical and Molecular Medicine, NTNU - Norwegian University of Science and Technology, Trondheim, Norway; 2 Department of Neuromedicine and Movement Science, NTNU - Norwegian University of Science and Technology, Trondheim, Norway; 3 Department of Rheumatology, St. Olavs University Hospital, Trondheim, Norway; 4 Department of Immunology and Transfusion Medicine, St. Olavs University Hospital, Trondheim, Norway; 5 Department of Clinical and Molecular Medicine, St. Olavs University Hospital, Lab Center 3 East, 7006 Trondheim, Norway

**Keywords:** Psoriatic arthritis, Physical activity, Body composition, Epidemiological study

## Abstract

The associations of physical activity (PA) with body composition among persons with psoriatic arthritis (PsA) are not well described. The objective was to investigate associations of PA with visceral fat mass and percentage body fat in persons with PsA of different age groups. Persons with PsA (CASPAR criteria, n = 356), and controls (n = 47,470) from the Trøndelag Health Study (HUNT4, 2017–2019) were included. Visceral fat mass and percentage body fat measured using bioelectrical impedance were primary outcomes in multivariable linear regression analysis. PsA, PA (questionnaire data), and age were explanatory variables, with adjustment for sex, smoking, heart disease, lung disease, and height. An interaction term between PsA and age was included in both models. Persons with PsA had altered body composition, including higher visceral fat mass and percentage body fat, especially those < 40 years of age (p ≤ 0.01). Moderate or high PA was associated with significantly lower values of the primary outcomes. Differences were Moderate compared to low PA: 1.4 kg (95% CI 1.3, 1.5 kg) lower visceral fat mass, and 2.0% (95% CI 1.8, 2.1) lower percentage body fat. Differences were High compared to low PA: 3.2 kg (95% CI 3.1, 3.3) lower visceral fat mass and 5.0% (95% CI 4.8, 5.1%) lower percentage body fat. Persons with PsA had higher visceral fat mass and percentage body fat, especially if < 40 years, and PA was associated with lower values of both endpoints. Changes of body composition in persons with PsA may influence important health outcomes and should be addressed in clinical practice.

**Supplementary Information:**

The online version contains supplementary material available at 10.1007/s00296-023-05348-9.

## Introduction

Psoriatic arthritis (PsA) is an autoimmune, inflammatory disease characterized by skin and joint involvement. The prevalence of PsA among persons with psoriasis is estimated to 15–30% [1, 2]. Clinical manifestations include peripheral and axial arthritis, enthesitis (inflammation at the sites where ligaments and tendons attach to bones), and dactylitis (inflammation of an entire digit) [3]. As PsA is classified as a spondyloarthropathy, it is also associated to uveitis and colitis. Apart from these manifestations, PsA is associated with comorbidities including the metabolic syndrome, diabetes, obesity, hyperlipidemia, hypertension, and insulin resistance, which significantly increase the risk of cardiovascular disease (CVD) morbidity and mortality [4–9]. Studies suggest that persons with PsA are less physically active [10] and have high prevalence of obesity [11] compared to the general population. Both low physical activity (PA) and obesity are known risk factors of CVD. Moreover, obesity increases the risk of developing PsA [12, 13].

Because of the high prevalence of obesity in persons with PsA, there has been an increasing interest in investigating their body composition. A systematic review showed that persons with PsA have an altered body composition, including higher visceral fat mass and percentage body fat compared to the general population [14]. Adipose tissue is a hormonally active organ, secreting adipokines, including tumor necrosis factor-α (TNF-α), leptin, resistin, and IL-6, which promote systemic inflammation and increase the risk of several pathophysiological conditions [15–17]. Having higher visceral fat mass is associated with increased risk of the metabolic syndrome [18], type 2 diabetes [19], CVD [20], non-alcoholic fatty liver disease [21], different types of cancer [22–24] as well as all-cause mortality [25], whereas having less visceral fat mass is associated with favorable health outcomes [26, 27].

Since body composition is modifiable in clinical practice, it is important to design proper interventions that could reduce visceral fat mass and percentage body fat in persons with PsA.

One such non-invasive intervention is PA. Although PA has well-established health benefits, its effects on body composition in persons with PsA is less investigated. A randomized controlled trial of 61 persons with PsA showed reduction of visceral fat after performing high-intensity interval training for 11 weeks [28]. However, because of the small sample size and lack of other studies, the generalizability of these results is not established.

We therefore performed a large population-based study aimed at investigating the relationship between visceral fat mass and percentage body fat, respectively, with PA in persons with PsA compared to controls. We also aimed to determine age-related differences of visceral fat mass and percentage body fat in persons with PsA and controls. Our hypothesis was that increased PA was associated with reduced visceral fat mass and percentage body fat, and that the alteration of body composition develops early in life, thus contributing to the comorbidity burden among persons with PsA. Furthermore, we performed an exploratory analysis to investigate the association between waist circumference and visceral fat mass. The exploratory aim was to evaluate whether waist circumference measurements could be used to estimate the amount of visceral fat mass in clinical practice.

## Methods

This retrospective analysis of cross-sectional case–control data utilized data from the fourth survey of the Trøndelag Health Study (HUNT4, 2017–2019) [29]. The present study is part of HuLIAS (HUNT Longitudinal Inflammatory Arthritis Study).

### Patients

**Fig. 1:**
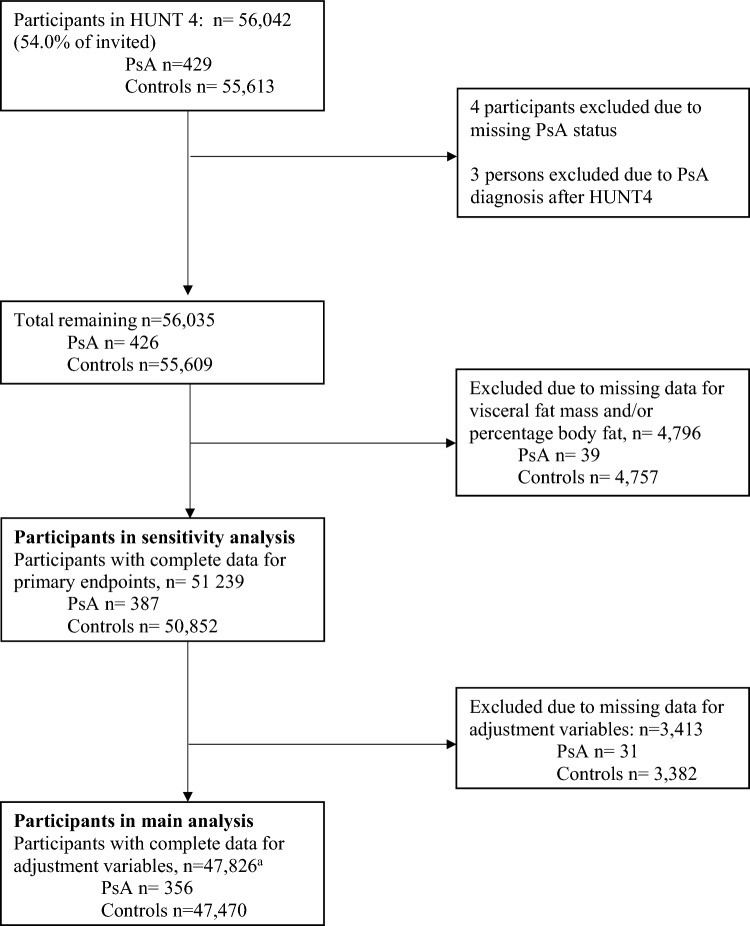
Participant inclusion and exclusion to the present study. ^a^missing data for sex n = 0, smoking n = 275, lung diseases n = 1728, heart disease n = 1859 and physical activity index n = 1437

The study included persons with PsA (n = 356), and controls (n = 47,470) with complete data for relevant variables in HUNT4. The inclusion criterion was participation in HUNT4. The exclusion criteria were missing information on PsA status or being given a PsA diagnosis within 1 year following HUNT4 participation, missing data for visceral fat mass and/or percentage body fat, and missing data for adjustment variables in the multivariable analysis, as detailed in Fig. 1. To diagnose PsA, data from HUNT4 were merged with medical records from two local hospitals, Levanger and Namsos hospitals, as well as the university hospital in Trondheim, St. Olavs Hospital. The medical records of persons with a probable PsA diagnosis were evaluated by an experienced senior rheumatologist (MH) using the ClASsification for Psoriatic ARthritis (CASPAR) criteria [30].

### Main outcome variable

The main outcome variables were visceral fat mass in kg and percentage body fat, measured in HUNT4 with bioelectrical impedance analysis with eight tactile electrodes (InBody770; Biospace, Tokyo, Japan). The device is based on the 4-compartment model (total body water, proteins, minerals, and body fat) and uses 6 frequencies (1, 5, 50, 250, 500, and 1000 kHz).

### Study factors

Explanatory variables of the models included PsA (yes/no), age (categorized into 3 categories: < 40, 40–59 and > 60 years, because of a non-linear association of age with the primary endpoints), and PA quantified based on self-reported questionnaire data. As in previous surveys, HUNT4 included 3 questions regarding PA, covering frequency (categories ranging from “never” to “almost every day”), duration (categories ranging from “ < 15 min” to “ > 1 h per session”), and intensity (categories ranging from “take it easy” to “push near exhaustion”) [31]. The replies were aggregated in two alternative ways: (1) using a summary index developed in the HUNT fitness sub-study with weighting of the responses [31], and categorizing the index values as low, moderate, or high PA, or (2) according to whether participants fulfilled the well-supported recommendations from the American College of Sports Medicine/American Heart Association for aerobic PA for healthy adults [32]. The recommendations are moderate intensity PA ≥ 30 min on five days a week or vigorous intensity aerobic PA ≥ 20 min on three days a week, or combinations to meet these criteria.

### Other variables

The statistical models for visceral fat mass and percentage body fat were adjusted for predefined variables influencing both PA and the primary endpoints, known from the literature and available in HUNT4. Adjustment variables included sex, self-reported smoking status (present, former, or never smoker), heart disease (yes/no, yes = myocardial infarction or heart failure), lung disease (yes/no, yes = chronic obstructive lung disease or asthma), and height to account for differences in body size.

### Procedures

HUNT is an open population-based cohort study carried out in the northern part of Trøndelag county, Norway [29]. All inhabitants $\ge$ 20 years of age were invited to participate. The fourth survey (HUNT4, 2017–2019, n = 56,042, i.e. 54% of those invited) consisted of questionnaires, interviews, clinical measurements, and analysis of blood samples. Participants in HUNT gave written informed consent. The study was approved by the Regional Committee for Medical and Health Research Ethics, Northern Norway (#11926) on 4/25/2019. The study was performed in accordance with the Helsinki Declaration.

### Statistical analysis

The distribution of the data was evaluated using histograms. Data are given as number with frequencies or means with standard deviation. Persons with PsA and controls were compared using Pearson’s chi-squared test, the Mann Whitney Wilcoxon Test, or the t-test depending on the distribution of the variable.

Associations of the explanatory variables with the endpoints were analyzed with multivariable linear regression. In the models, PA was evaluated as low, moderate, or high based on the index calculated from self-reported PA frequency, duration, and intensity, because this variable gave better model fit. To investigate whether body composition in persons with PsA compared to controls differed according to age, an interaction term between PsA and age group was tested in the models. We used robust estimation of standard errors. Assumptions of multivariable linear regression and model fit, including linear relationships between predictors and the outcomes and normality of the residuals were evaluated using plots. As a sensitivity analysis to evaluate whether the results were biased due to analysis of complete cases for the adjustment variables, multiple imputation of missing data was performed. We used chained equations (n = 150 data sets), assuming “missing at random”.

To investigate the correlation between visceral fat mass and waist circumference, a simple linear regression analysis with visceral mass (dependent variable) and waist circumference (independent variable) was performed. Statistical analyses were performed using Stata (v. 16.1 StataCorp. College Station, TX, USA). P-values < 0.05 were considered statistically significant.

## Results

The study included n = 356 persons with PsA and n = 47,470 controls. Participant characteristics are given in Table 1. Persons with PsA were older and more often unemployed at the time of research compared to controls. They also had significantly higher prevalences of diabetes, cancer, and hypertension, as well as higher levels of serum triglycerides and cholesterol. Persons with PsA were less physically active according to the recommendations for PA (Table 2) and were more often smokers. They had also higher weight, body mass index, visceral fat mass, percentage body fat, and waist-hip ratio.

**Table 1:** Demographic and clinical characteristics

Variable	PsA, n = 356	Controls, n = 47,470	p-value
Age years, mean (SD)	58 (12)	53 (17)	p < 0.001
Age at PsA diagnosis years, mean (SD)	46 (11)	–	
Years with PsA diagnosis at time of study participation^a^		–	–
9 years or less, n (%)	20 (9)		
10–19 years, n (%)	115 (54)		
20 years and above, n (%)	78 (37)		
Male, n (%)	200 (56)	25,731 (54)	p = 0.46
Diabetes^b^, n (%)	35 (10)	2440 (5)	p < 0.001
Heart disease^c^, n (%)	19 (5)	1753 (4)	p = 0.10
Hypertension^d^, n (%)	169 (47)	17,188 (36)	p < 0.001
Respiratory disease^e^, n (%)	50 (14)	6200 (13)	p = 0.58
Hypercholesterolemia, n (%)	79 (22)	9443 (20)	p = 0.30
Cancer, n (%)	35 (10)	3281 (7)	p = 0.033
Serum triglycerides mmol/L, mean (SD)	1.9 (1.2)	1.6 (1.0)	p < 0.001
HDL cholesterol mmol/L, mean (SD)	1.4 (0.4)	1.4 (0.4)	p = 0.98
Systolic blood pressure mmHg, mean (SD)	130 (18)	128 (18)	p = 0.033
Diastolic blood pressure, mmHg, mean (SD)	74 (10)	73 (10)	p = 0.25
CRP mg/L, mean (SD)	4 (6)	3 (5)	p < 0.001
eGFR ml/min/1.73m^2^, mean (SD)	91 (16)	93 (19)	p = 0.034
Alcohol (twice a week or more), n (%)	78 (22)	9623 (20)	p = 0.45
Smoking status, n (%)			p < 0.001
Present	58 (16)	4688 (10)	
Former	201 (57)	21,567 (45)	
Never	97 (27)	21,215 (45)	
Education (approved apprenticeship, college or higher), n (%)	195 (55)	29,711 (63)	p = 0.002
Unemployed at the time of HUNT4 participation, n (%)	163 (46)	16,473 (35)	p < 0.001

*PsA* psoriatic arthritis, *HDL* high-density lipoprotein, *CRP* C-reactive protein, *eGFR* estimated glomerular filtration rate, *HUNT4* the 4th survey of the longitudinal population-based Trøndelag Health Study

^a^143 persons with PsA were missing the year of diagnosis

^b^Diabetes: self-reported diabetes

^c^Heart disease: myocardial infarction or heart failure; self-reported

^d^Hypertension: systolic blood pressure $\ge$ 140 mm Hg and/or a diastolic blood pressure $\ge$ 90 mm Hg and/or the use of antihypertensive medication

^e^Respiratory disease: self-reported asthma and/or chronic obstructive pulmonary disease

**Table 2:** Body composition and physical activity

Variable	PsA, n = 356	Controls, n = 47,470	p-value
Height cm, mean (SD)	171 (9)	171 (9)	p = 0.65
Weight kg, mean (SD)	84 (17)	80 (16)	p < 0.001
Body mass index kg/m^2^, mean (SD)	28.6 (4.7)	27.2 (4.7)	p < 0.001
Waist-hip ratio, mean (SD)	0.99 (0.1)	0.96 (0.1)	p < 0.001
Body fat mass kg, mean (SD)	28.8 (11)	25.2 (11)	p < 0.001
Percentage body fat, mean (SD)	33.8 (8.7)	30.9 (9.4)	p < 0.001
Visceral fat mass kg, mean (SD)	13.7 (5.5)	11.7 (5.5)	p < 0.001
Physical activity index			p = 0.14
Low	204 (57)	24,949 (53)	
Moderate	104 (29)	14,636 (31)	
High	48 (14)	7885 (16)	
Did not meet the recommended level of physical activity^a^, n (%)^2^	201 (61)	25,674 (56)	p = 0.047

*PsA* psoriatic arthritis

^a^Recommendations of physical activity for adults from the American College of Sports Medicine /American Heart Association [32]

The regression model for visceral fat mass (R^2^ = 0.15) showed that having a diagnosis of PsA was associated with a mean increase in visceral fat mass of 2.0 kg (95% CI 1.2, 2.8 kg). Table 3 gives the coefficients for variables in the model. Moderate and high PA were significantly associated with lower visceral fat mass both for persons with PsA and controls. Moderate PA was associated with 1.4 kg (95% CI 1.3, 1.5 kg), and high PA with 3.2 kg (95% CI 3.1, 3.3) lower visceral fat mass compared to low PA.

**Table 3:** Regression analysis of visceral fat mass and percentage body fat

Variable	Visceral fat mass (kg)		Percentage body fat	
	Regression coefficient (Standard error)	p-value	Regression coefficient (Standard error)	p-value
PsA	4.0 (1.1)	p < 0.001	5.2 (1.9)	p = 0.006
Age group				
20–39 years	Reference		Reference	
40–59 years	2.1 (0.1)	p < 0.001	3.1 (0.1)	p < 0.001
60 years and above	3.1 (0.1)	p < 0.001	4.5 (0.1)	p < 0.001
Interaction between PsA and age group^a^	− 2.6 (1.4)	p = 0.026	− 3.8 (1.8)	p = 0.013
Female sex	− 3.3 (0.1)	p < 0.001	− 7.6 (0.1)	p < 0.001
Physical activity index				
Low	Reference		Reference	
Moderate	− 1.4 (0.1)	p < 0.001	− 2.0 (0.1)	p < 0.001
High	− 3.2 (0.1)	p < 0.001	− 5.0 (0.1)	p < 0.001

The models were adjusted for sex, smoking status, heart disease, lung disease, and height

*PsA* psoriatic arthritis

^a^Age groups were coded as 0 = 20–39 years, 1 = 50–59 years, and 2 = 60 years and above

The model for percentage body fat (R^2^ = 0.38) showed that a diagnosis of PsA was associated with a mean increase in percentage body fat of 2.7% (95% CI 1.6, 3.8, Table 3) Moderate or high PA was significantly associated with lower values of percentage body fat both for persons with PsA and controls. Moderate PA was associated with 2.0% (95% CI 1.8, 2.1) and high PA was associated with 5.0% (95% CI 4.8, 5.1%) lower percentage body fat compared to low PA.

**Fig. 2:**
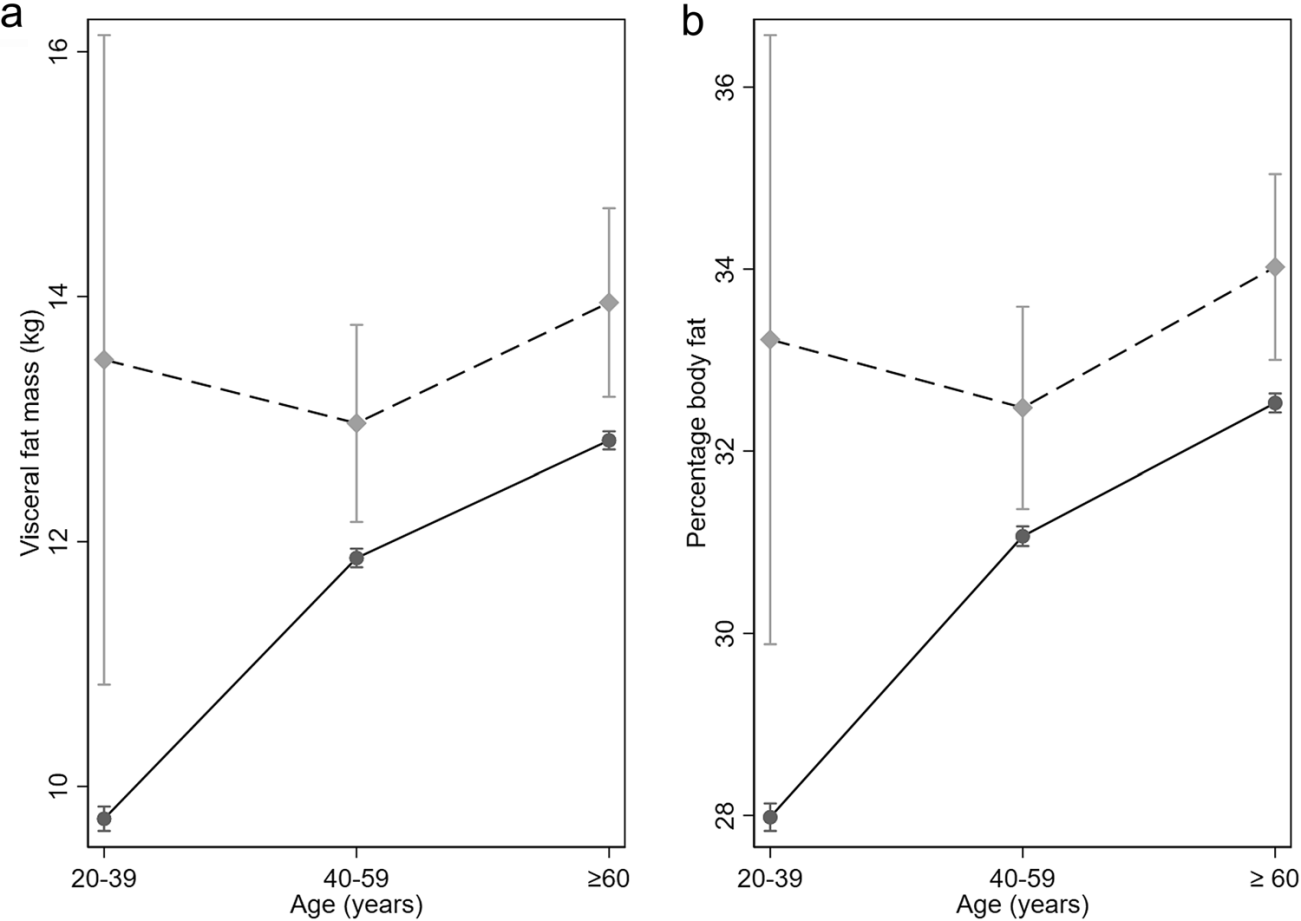
Interaction effects with age. **a** Visceral fat mass in participants with psoriatic arthritis and controls in different age groups (mean with 95% confidence interval). **b** Percentage body fat in participants with psoriatic arthritis and controls in different age groups (mean with 95% confidence interval)

The interaction term between PsA and age groups was statistically significant for both models (visceral fat mass: p = 0.026, percentage body fat: p = 0.013). The difference in visceral fat mass in individuals with PsA compared to controls of the same age group was relatively larger in persons < 40 years (visceral fat mass difference in persons < 40 years: 4.9 kg (95% CI 1.9, 7.9 kg) vs. 1.2 kg (95% CI 0.1–2.4) in age group 40–69 years and 1.4 kg (95% CI 0.2, 2.5 kg) in age 60 years and above (Fig. 2a). The difference in percentage body fat was also higher in persons with PsA < 40 years (percentage body fat difference in persons < 40 years, 5.2% (1.9, 8.5) vs. 1.2 kg (95% CI 0.1–2.4) in age group 40–69 years and 1.4 kg (95% CI 0.2, 2.5 kg) in age 60 years and above (Fig. 2b).

Results from the sensitivity analysis in the complete dataset following multiple imputation of missing data gave very similar results as the main analysis and thus confirmed that the results were not biased by excluding the participants with missing data.

The simple linear regression model of visceral fat mass and waist circumference was statistically significant (R^2^ = 0.75, p < 0.001), illustrating that waist circumference measurements could estimate the values of visceral fat mass. However, the estimation was not precise as seen in a scatterplot (Online Resource 1—Scatterplot of waist circumference and visceral fat mass). The mean difference between the predicted and observed values was 2.8 kg.

## Discussion

The present study showed that moderate and high PA were significantly associated with lower visceral fat mass and percentage body fat both in persons with PsA and controls. However, PsA was associated with higher values of visceral fat mass and percentage body fat compared to controls. The greatest differences compared to controls of the same age group were seen among persons with PsA < 40 years of age.

The present study strengthens the evidence of body composition alterations in persons with PsA and supports previous studies [14, 28, 33–38]. A few studies with small sample sizes did not find these associations [39, 40]. The reasons for the body composition changes are thought to be complex involving genetic disposition, disease pathophysiology, medications, as well lifestyle factors such as PA and diet [14, 41–43].

Our study further showed that persons with PsA < 40 years had the highest values for visceral fat mass and percentage body fat compared to controls of the same age group. Since these persons are expected to live longer, the negative impacts of visceral fat mass and percentage body fat may compound over several decades, contributing the comorbidity burden observed among persons with PsA. The reasons for the body composition changes in persons with PsA < 40 years are not known. However, a population-based study in Sweden showed that younger women with spondylarthritis including PsA tended to be less compliant with the WHO recommendations for PA [44]. This suggests that low PA may be a potential explanatory factor. Longitudinal studies are therefore needed to investigate the reasons and impacts of the body composition changes in young persons with PsA.

The present study also showed that persons with PsA were less physically active according to the recommendations of PA for adults [32]. These findings are in accordance with the results of many studies that demonstrate low PA in persons with PsA [10, 41]. Systemic inflammation is regarded as a link between PsA and CVD [18], and PA is acknowledged to reduce both chronic inflammation [45] and CVD [46] in the general population. Therefore, the low PA levels in persons with PsA may lead to increased CVD morbidity and mortality and should be addressed in clinical settings.

Moderate or high PA was associated with reduced levels of visceral fat mass and percentage body fat both in persons with PsA and controls. Because of the cross-sectional design of the present study, it was not possible to determine causality. However, several studies in the general population [47], as well as a small study of persons with PsA [28] indicated a causal relationship. In these studies, higher PA levels led to favorable changes in body composition in terms of reduced visceral fat mass and overall body fat. To our knowledge, the present study is the first that examined the association of PA with visceral fat mass and percentage body fat in a population-based setting.

The exploratory analysis showed that waist circumference was significantly positively correlated with visceral fat mass. In clinical practice, measuring waist circumference is relatively easy even if accuracy may depend on body shape, and may estimate the amount of visceral fat mass. However, our study clearly demonstrated that these measurements are not precise, and their limitations should be acknowledged in clinical practice.

The study has several strengths. Bioelectrical impedance analysis was used to measure body composition, which is more precise than anthropometric measurements such as BMI, skinfold thickness, waist and hip circumference, and hip-waist ratio. Body composition can be measured with several non-anthropometric methods including bioelectrical impedance analysis, dual-energy X-ray absorptiometry, and computer tomography. As of today, there is no consensus on the optimal advanced assessment method of body composition among persons with PsA [48], giving challenges both in research and clinical practice [14]. Therefore, it is important to standardize the methods to be able to compare different data regarding body composition.

Another strength of the study was the large number of participants in a population-based study, as well as validated PsA diagnoses. The findings of the study are therefore more likely to be applicable in the general population.

The study also has limitations. The use of self-reported PA may lead some persons with PsA to overestimate the intensity of PA levels because they experience joint pain or fatigue. Another limitation of the study is that bioelectrical impedance analysis may underestimate the percentage body fat it in obese participants [49]. This may apply to persons with PsA in our study since they had significantly higher weight than controls.

In conclusion, the present study showed that moderate and high PA was associated with lower values of visceral fat mass and percentage body fat both in persons with PsA and controls. Persons with PsA had higher visceral fat mass and percentage body fat compared to controls of their age group, especially persons with PsA < 40 years. They were also less compliant with the recommended PA levels. The study emphasizes the importance of screening the body composition of persons with PsA and evaluate factors contributing to these changes. Low PA levels among persons with PsA may lead to changes in body composition and influence important health outcomes, and should be addressed in clinical settings.

## Supplementary Information

Below is the link to the electronic supplementary material.

Supplementary file1 (PDF 146 KB)

## Data Availability

Data from HUNT are available upon reasonable request from the HUNT Research Centre (www.ntnu.edu/hunt/data), following approval from the Regional Ethics Committee. However, restrictions apply to the availability of the data for the present paper, which were used under license for the current study and are not publicly available in accordance with Norwegian law.
