# Supplementary material for: High physical activity in persons with psoriatic arthritis is associated with reduced visceral fat mass and percentage body fat: the Trøndelag Health study
Source: Rheumatol Int. 2023 Jun 5;43(9):1685–93. doi: 10.1007/s00296-023-05348-9 (PMC10348995; doi:10.1007/s00296-023-05348-9)

Osman AA, Hoff M, Videm V: High physical activity in persons with psoriatic arthritis is associated with reduced visceral fat mass and percentage body fat - The Trøndelag Health study

Rheumatology International

Corresponding author: Vibeke Videm, Department of Clinical and Molecular Medicine, NTNU – Norwegian University of Science and Technology and Department of Immunology and Transfusion Medicine, St. Olavs University Hospital, Trondheim, Norway. E-mail: vibeke.videm@ntnu.no

Online Resource 1: Scatterplot of waist circumference and visceral fat mass

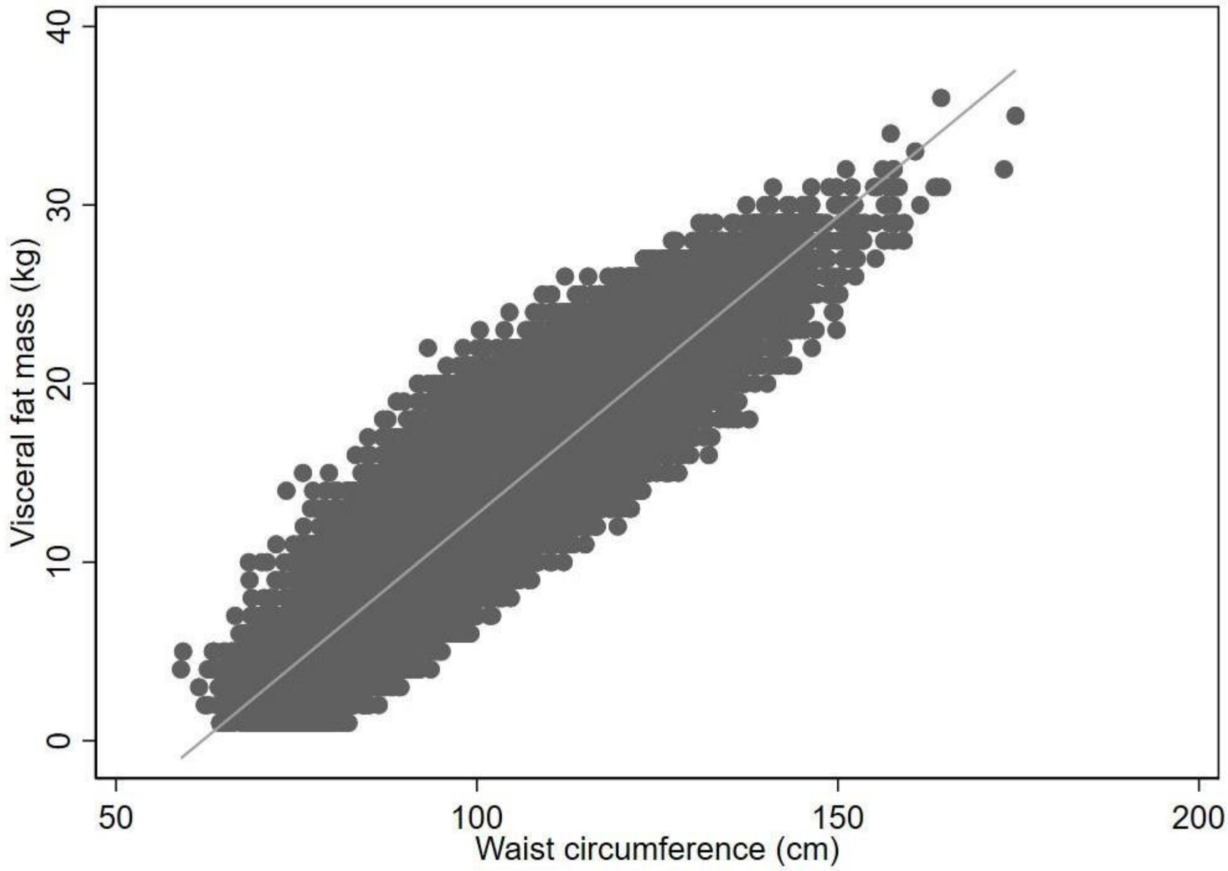

Supplement: Supplementary file 1 — Supplementary file1 (PDF 146 KB) [file 296_2023_5348_MOESM1_ESM.pdf]
